# Diallyl Trisulfide Enhances Doxorubicin Chemosensitivity by Inhibiting the Warburg Effect and Inducing Apoptosis in Breast Cancer Cells

**DOI:** 10.7150/jca.113578

**Published:** 2025-07-11

**Authors:** Chun-Ming Chang, Wei-Jan Wang, Thomas G. Mhone, Wen-Kun Ho, Chih-Jung Chen, Shawn Shang-Chuan Ng, Chi-Cheng Li, Chia-Hua Kuo, Chih-Yang Huang, Wei-Wen Kuo

**Affiliations:** 1 Department of General Surgery, Hualien Tzu Chi Hospital, Buddhist Tzu Chi Medical Foundation, Hualien, Taiwan.; 2 Institute of Medical Sciences, Tzu Chi University, Hualien, Taiwan.; 3 Department of Biological Science and Technology, China Medical University, Taichung 40676, Taiwan.; 4 Research Centre for Cancer Biology, China Medical University, Taichung 40676, Taiwan.; 5 Graduate Institute of Biomedicine, China Medical University, Taichung, Taiwan.; 6 Ph.D. Program for Biotechnology Industry, China Medical University, Taichung 406, Taiwan.; 7 Division of Breast Surgery, Department of Surgery, China Medical University Hospital, Taichung 40447, Taiwan.; 8 Department of Hematology and Oncology, Hualien Tzu Chi Hospital, Buddhist Tzu Chi Medical Foundation, Hualien, Taiwan.; 9 School of Medicine Tzu Chi University, 701, Section 3, Chung-Yang Road, Hualien 97004, Taiwan.; 10 Center of Stem Cell & Precision Medicine, Hualien Tzu Chi Hospital, Buddhist Tzu Chi Medical Foundation, Hualien, Taiwan.; 11 Laboratory of Exercise Biochemistry, University of Taipei, Taipei, Taiwan.; 12 Department of Kinesiology and Health Science, College of William and Mary, Williamsburg, VA, USA.; 13 Cardiovascular and Mitochondria related diseases research center, Hualien Tzu Chi Hospital, Hualien 970, Taiwan.; 14 Department of Biotechnology, Asia University, Taichung 413, Taiwan.; 15 Center of General Education, Buddhist Tzu Chi Medical Foundation, Tzu Chi University of Science and Technology, Hualien 970, Taiwan.; 16 Department of Medical Research, China Medical University Hospital, China Medical University, Taichung 404, Taiwan.; 17 School of pharmacy, China Medical University, Taichung, Taiwan.

**Keywords:** diallyl trisulfide, Warburg effect, chemosensitivity, LDHA, apoptosis

## Abstract

Breast cancer is the leading cause of cancer-related mortality among women. Doxorubicin (DOX) is the major chemotherapeutic agent for breast cancer treatment, but its efficacy is hindered by chemoresistance and dose-dependent toxicity. Overcoming these challenges requires novel therapeutic strategies that enhance DOX sensitivity while minimizing its adverse effects. Diallyl trisulfide (DATS), a natural organosulfur compound derived from garlic, has demonstrated anticancer potential, yet its role in enhancing DOX chemosensitivity remains unclear. The anticancer potential of the combination treatment was investigated using MTT, glucose uptake, and lactate production assays, Western blot, flow cytometry, TUNEL staining, transfection, and an *in vivo* orthotopic tumor model in NOD/SCID mice. DATS and DOX combination treatment synergistically inhibited the viability of breast cancer cell lines. The combination treatment significantly reduced glucose uptake and lactate production while downregulating key glycolytic regulators, including GLUT1, LDHA, and HIF-1α. These metabolic alterations were associated with enhanced apoptosis, as evidenced by elevated expression of Bax, cleaved caspase-3, and PARP1, along with downregulation of anti-apoptotic proteins Bcl-2 and Bcl-xL. TUNEL and Annexin V-FITC/PI assays further confirmed apoptosis induction in response to combination therapy. *In vivo* studies using an orthotopic MDA-MB-231 xenograft model revealed that DATS and DOX combination treatment significantly suppressed tumor growth while reducing systemic toxicity, as indicated by stable body weight and minimal adverse effects. Overall, our findings show that DATS enhances DOX sensitivity by inhibiting the Warburg effect and promoting apoptosis in breast cancer cells. These findings suggest that the DOX-DATS combination represents a promising strategy to improve chemotherapeutic efficacy in breast cancer.

## 1. Introduction

Breast cancer is the most frequently diagnosed malignancy and the leading cause of cancer-related mortality among women worldwide ^1,2.^ Despite significant advancements in therapeutic strategies, the effectiveness of conventional chemotherapy remains limited by the emergence of drug resistance, tumor heterogeneity, and systemic toxicity 3,4. Doxorubicin (DOX), an anthracycline chemotherapeutic agent, is widely used for breast cancer treatment due to its potent anti-proliferative and pro-apoptotic properties 5. However, the clinical utility of DOX is often compromised by intrinsic and acquired chemoresistance, as well as dose-dependent toxicity, particularly cardiotoxicity 6-8. These limitations highlight the urgent need for novel therapeutic strategies to enhance DOX chemosensitivity while minimizing its adverse effects.

One of the key mechanisms underlying chemoresistance in breast cancer is metabolic reprogramming, particularly the Warburg effect, wherein cancer cells preferentially utilize aerobic glycolysis over oxidative phosphorylation to sustain rapid proliferation and survival 9,10. This metabolic adaptation is driven by increased glucose uptake and lactate production, facilitated by key glycolytic regulators such as glucose transporter 1 (GLUT1), lactate dehydrogenase A (LDHA), and hypoxia-inducible factor-1 alpha (HIF-1α)^11^-^16^. This metabolic shift not only provides cancer cells with a bioenergetic advantage but also confers resistance to apoptosis and increases drug efflux, thereby reducing the efficacy of chemotherapeutic agents 17. Targeting the Warburg effect has therefore emerged as a promising strategy for enhancing the therapeutic potential of conventional chemotherapies and overcoming drug resistance.

Diallyl trisulfide (DATS), a bioactive organosulfur compound derived from garlic (Allium sativum), has garnered significant attention for its broad-spectrum anticancer properties. Previous studies have demonstrated that DATS exerts its anti-tumor effects by modulating key cellular processes, including oxidative stress, apoptosis, cell cycle arrest, and metabolic reprogramming. However, the impact of DATS on DOX sensitivity and its ability to reverse the Warburg effect in breast cancer cells remains poorly understood. In this study, we investigate the potential of DATS to enhance the chemosensitivity of breast cancer cells to DOX by targeting metabolic reprogramming and promoting apoptosis.

## 2. Material and Methods

### 2.1 Cell Culture

The MDA-MB-231, MCF7, MCF10, Clone 9, BEAS-2B, and AC16 cell lines were obtained from the American Type Culture Collection (ATCC, Virginia, USA). All cell lines, except AC16, were cultured in high-glucose Dulbecco's Modified Eagle Medium (DMEM) supplemented with 3.7 g/L sodium bicarbonate, 1% penicillin-streptomycin (PS), and 10% fetal bovine serum (FBS). AC16 cells were cultured in DMEM/F-12 medium containing 1.2 g/L sodium bicarbonate, 1% PS, and 12% FBS. All cell cultures were maintained in a humidified incubator at 37°C with 5% CO₂. When cell confluency reached 70-80%, cultures were either passaged to maintain proliferation or seeded for experimental assays.

### 2.2 Drugs

Diallyl trisulfide (CAS No. 250-87-5) was procured from MedChemExpress (USA). Doxorubicin (CAS No. 25316-40-9) was obtained from Cayman Chemical (USA). LDHA siRNA was purchased from Sigma-Aldrich (USA).

### 2.3 MTT cell viability assay

Cell viability was evaluated using the MTT assay. Briefly, normal and cancer cells seeded in a 96 well plate were treated with various concentrations of DOX and DATS for 24 and 48 hours. Next, MTT solution (0.5 mg/mL) was added to each well and incubated for 2 hours at 37°C, followed by addition dimethyl sulfoxide (DMSO) to dissolve the formazan crystals. The Absorbance of the MTT solution was measured at 595 nm using a microplate reader (Molecular Devices SpectraMax® ABS Plus, USA). For combination treatments, cancer cells were first exposed to Dox for 24 hours, followed by addition of DATS for a 48-hour combined treatment. ComboSyn software was used to calculate combination index (CI). In our study, CI > 1 indicates antagonism, CI = 1 additive, and CI < 1 synergism 18,19.

### 2.4 Whole Cell Protein Lysate Extraction

Total protein extraction was performed using RIPA lysis buffer as previously described 18. Briefly, cells were washed with PBS, followed by the addition of RIPA buffer and incubation on ice for 30 minutes. After incubation, samples were centrifuged at 15,000 rpm for 30 minutes, and the supernatant containing the total protein was collected for western blot analysis.

### 2.5 Bradford protein assay

Protein concentrations in the lysates were quantified using the Bradford Protein Assay, following the manufacturer's instructions. Briefly, 20 µL of each protein sample and serially diluted bovine serum albumin (BSA) standards (1 mg/mL) were incubated with Coomassie Brilliant Blue dye. The absorbance of the resulting protein-dye complexes was measured at 595 nm using an ELISA reader. Protein concentrations were then calculated based on a standard curve generated from the absorbance values of the BSA standards 18.

### 2.6 Immunoblotting

Protein lysates (30-50 μg) were boiled in 5x sample buffer for 5-10 minutes, then separated by 7-15% SDS-polyacrylamide gel electrophoresis (SDS-PAGE) and transferred onto polyvinylidene fluoride (PVDF) membranes. The membranes were then blocked with 5% non-fat milk for 1 hour to prevent non-specific antibody binding. After blocking, the membranes were incubated with specific primary antibodies overnight at 4°C. This was followed by incubation with secondary antibodies for 1 hour at room temperature. Protein bands were visualized using the Fuji LAS 3000 imaging system. 18,20.

### 2.7 Antibodies

The following antibodies were used in this study: from Cell Signaling Technology: Anti-LDHA (C4B5), Anti-Caspase 3, and Anti-Cleaved Caspase 3. From Santa Cruz Biotechnology: Anti-GLUT1 (A-4), Anti-Pyruvate Dehydrogenase, and Anti-β-actin (C4), Anti-HIF1α was obtained from Gentex. Secondary antibodies (anti-rabbit, anti-mouse, and anti-goat) were sourced from Santa Cruz Biotechnology.

### 2.8 Glucose uptake flow cytometry analysis

A glucose uptake assay (Abcam, USA) was performed following the manufacturer's protocol. Breast cancer cells were treated with DATS and DOX in DMEM containing 0.5% fetal bovine serum (FBS) for 24 hours. After treatment, the medium was replaced with glucose uptake medium, which included 2-NBDG reagent, a glucose uptake enhancer, DATS, and DOX in DMEM with 0.5% FBS. Cells were incubated for 30 minutes at 37°C in a 5% CO₂ atmosphere. Post-incubation, the cells were harvested by trypsinization, centrifuged, and resuspended in 400 µL of 1X analysis buffer. Glucose uptake was then quantified using flow cytometry with a 488 nm excitation laser on a BD FACSLyric™ instrument (BD Biosciences, San Jose, CA) 20.

### 2.9 Annexin V and PI double staining flow cytometry analysis

Apoptosis was evaluated using FITC Annexin V and propidium iodide (PI) (BD Biosciences, USA) double staining, following the manufacturer's protocol. Briefly, breast cancer cells were trypsinized, centrifuged, and resuspended in binding buffer. To each sample, 5 µL of FITC Annexin V and 5 µL of PI were added, followed by flow cytometric analysis using the BD FACSLyric™ instrument. Data were analyzed with FlowJo software (version 7.6.1), and apoptosis rates were quantified as the percentage of dead cells in the drug-treated groups compared to the control group. 18,19.

### 2.10 Lactate production assay

Lactate production was assessed using a Lactate Assay Kit (Abcam, USA) following the manufacturer's instructions. Breast cancer cells were collected via trypsinization and resuspended in lactate assay buffer. The suspension was homogenized by vortexing and centrifuged at 16,000 rpm for 5 minutes at 4°C. The supernatants were then collected for analysis. In a 96-well plate, 50 µL of lactate reaction mix—containing Lactate Assay Buffer, Lactate Substrate Mix, and Lactate Enzyme Mix—was added to 50 µL of each sample and standards. The reaction mixture was incubated at room temperature for 30 minutes, followed by measuring absorbance at 450 nm using a microplate reader (Molecular Devices SpectraMax® ABS Plus, USA). Lactate concentration was calculated using a standard curve generated from the lactate standards 20.

### 2.11 Transfection assay

LDHA-siRNA was obtained from Sigma-Aldrich, and the jetPRIME transfection reagent was sourced from Polyplus Transfection (Illkirch, France). The assay was performed according to the manufacturer's instructions. MDA-MB-231 breast cancer cells seeded in 6-well plates were transfected using the jetPRIME transfection buffer for 18 hours. After incubation the cells were further treated with DOX for an additional 24 hours 21.

### 2.12 TUNEL staining

TUNEL staining (Sigma-Aldrich/Roche) was performed according to the manufacturer's protocol. Briefly, breast cancer cells grown on chamber slides were fixed with 4% cell-grade formaldehyde for 1 hour, followed by permeabilization with 0.2% Triton X-100 for 10 minutes. Then, the slides were incubated in TUNEL staining reaction mixture for 1 hour at 37°C in the dark. Finally, Fluoroshield with DAPI was used mount the slides followed by fluorescence microscopy (Olympus DP73 microscope (Olympus Corporation, Center Valley, PA, USA)) 18,22.

### 2.13 Animal model and treatments

The animal protocol was approved by the Laboratory Animal Service Center at China Medical University (Approval no. 2016-172-1), adhering to the principles of the 3Rs (Replacement, Reduction, and Refinement) and the Humane Care and Use of Laboratory Animals guidelines 18. Six-week-old Nonobese diabetic/severe combined immunodeficiency (NOD/SCID) mice, sourced from BioLASCO (Taiwan), were used for this study. The mice were housed under a 12-hour light/dark cycle at 25°C with standard rodent chow and water. Luciferase-expressing MDA-MB-231 cancer cells were orthotopically implanted into the fourth abdominal mammary fat pads. Mice were monitored weekly for general health, bioluminescence intensity, tumor volume, and body weight 23. Once tumors reached 500 mm³, the mice were randomized into five groups: normal control (n=3), vehicle control (n=3), Dox-treated (n=3), DATS-treated (n=3), and combination treatment (n=3). Doxorubicin (1 mg/kg) was administered intraperitoneally once a week for 4 weeks, while DATS (40 mg/kg) was given intratumorally twice a week for 4 weeks. When the tumor size reached 1,500 mm^3^, the mice were considered expired. At the end of the treatment period, the mice were euthanized, and tumors were collected for subsequent analyses.

### 2.14 LDHA-DATS molecular docking analysis

Molecular docking of DATS with LDHA was performed using SwissDock (http://www.swissdock.ch/), based on the EADock DSS engine. The crystal structure of human LDHA (PDB ID: 1i10) was retrieved from the Protein Data Bank. Protein structure preparation included removal of water molecules and addition of polar hydrogens. Docking was conducted using the "docking with attracting cavities" mode to focus the search within the active site. The DATS structure was optimized prior to docking, and the best-ranked pose was selected based on the AC Score and SwissParam Score. Visualization and analysis of binding interactions were performed using SwissDock NGL viewer.

### 2.15 Statistical analysis

Differences among multiple groups were analysed using ANOVA, followed by suitable post-hoc test. p-values of < 0.05 were considered statistically significant. All experimentations were performed in triplicates.

## 3. Results

### 3.1 Diallyl trisulfide demonstrates selective cytotoxicity in normal and cancer cells

**Figure 1:**
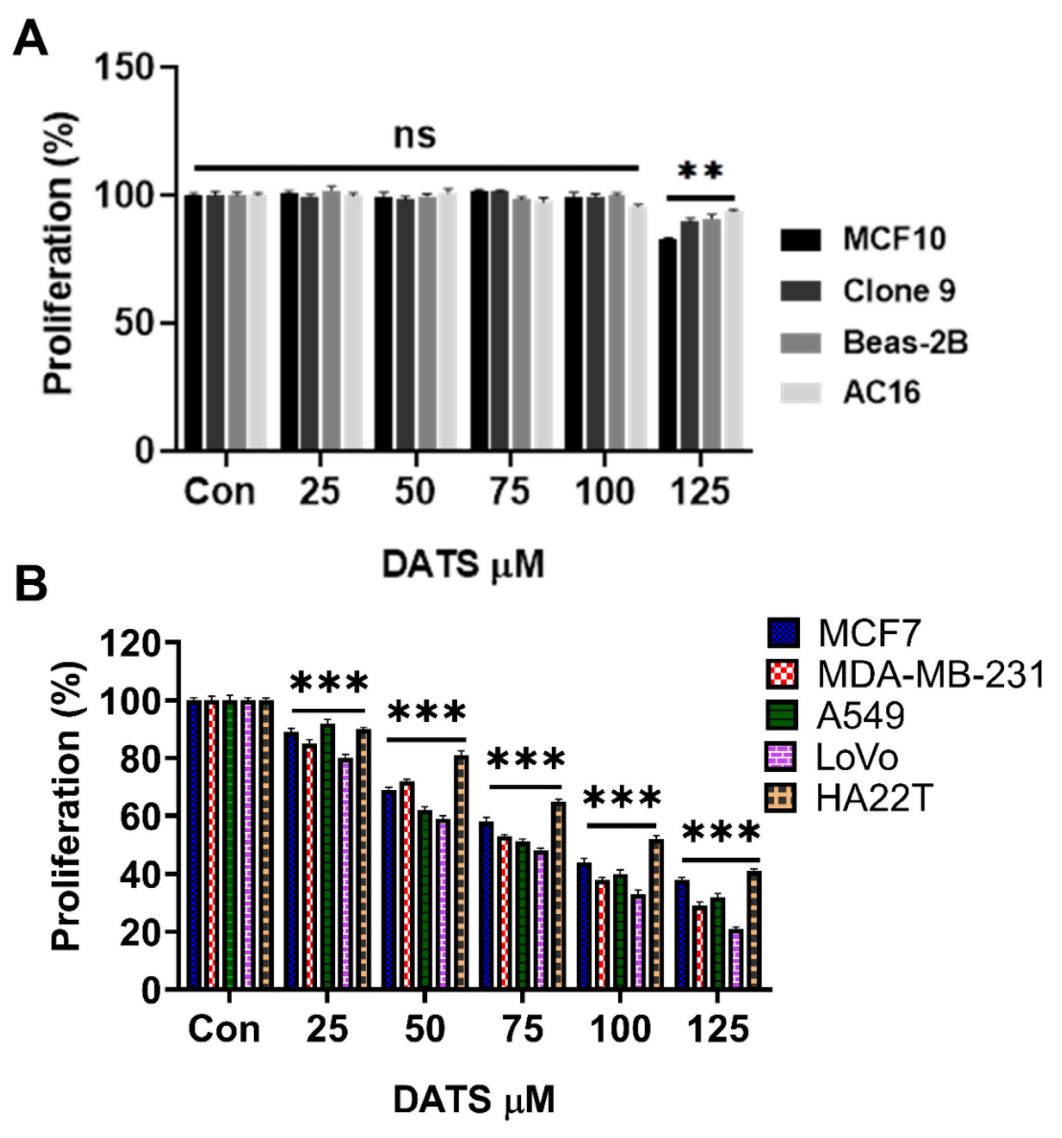
** Diallyl trisulfide demonstrates selective cytotoxicity in normal and cancer cells. (A)** Normal cell lines (MCF10, Clone 9, Beas-2B, and AC16) were treated with increasing concentrations of DATS for 24 hours followed by MTT cell viability assay. **(B)** Cancer cell lines (MCF7, MDA-MB-231, A549, LoVo, and HA22T) were exposed to the same DATS treatment as in **(A)**, followed by MTT cell viability assay. These results are expressed as a percentage of viable cells from treated groups compared with control. All experiments were conducted in triplicate, ns=not significant, **P < 0.001 and ***P < 0.0001.

First, the cytotoxicity of DATS was evaluated in normal cell lines derived from the breast (MCF10), liver (Clone 9), lung (Beas-2B), and heart (AC16). The cells were exposed to increasing concentrations of DATS (0, 25, 50, 75, 100, and 125 µM) for 24 hours, followed by the MTT cell viability assay. Our data revealed that DATS at 25 to 100 µM had minimal cytotoxic effects on all tested normal cell types, with cell viability remaining above 85% **(Figure 1A)**.

**Figure 2:**
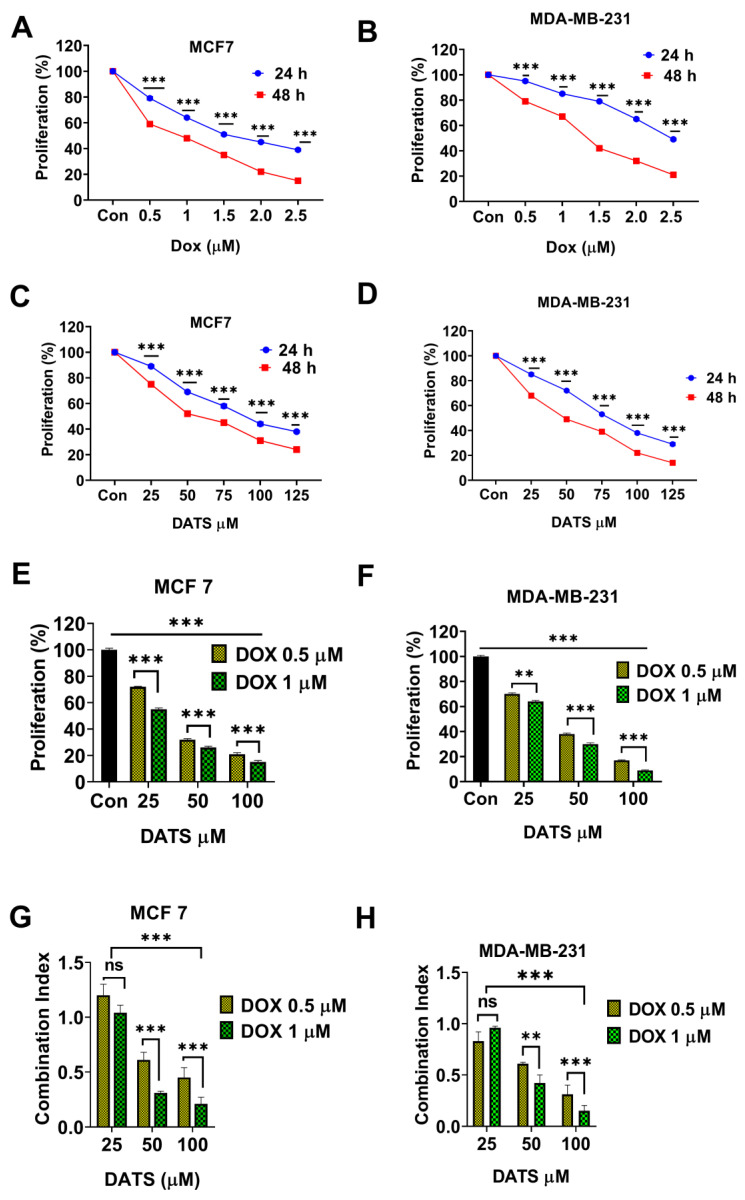
** Diallyl trisulfide synergistically enhances doxorubicin cytotoxicity in breast cancer cells. (A and B)** Breast cancer cells—(A) MCF7 and (B) MDA-MB-231—were treated with the indicated concentrations of DOX for 24 and 48 hours, followed by MTT cell viability assay. **(C and D)** Breast cancer cells —** (C)** MCF7 and **(D)** MDA-MB-231— were treated with the indicated concentrations of DATS for 24 and 48 hours, followed by the MTT cell viability assay. **(E and F)** Breast cancer cells— **(E)** MCF7 and **(F)** MDA-MB-231—were pre-treated with Dox (0.5 and 1 µM) for 24 hours, followed by the addition of DATS (50 and 100 µM) for a combined treatment period of 48 hours, followed by MTT cell viability assay. **(G and H)** Combination index (CI) calculation in **(G)** MCF7 and** (H)** MDA-MB-231 cells. The CI reflects the degree of drug-drug interactions; in this study, CIs <0.9 indicated synergism, CIs ranging from 0.9 to 1.1 indicated additive effects, and CIs >1.1 indicated antagonistic effects. These results are expressed as a percentage of viable cells from treated groups compared with control cells, ns= not significant, *P < 0.05, **P < 0.01, ***P < 0.001.

Subsequently, the same DATS doses were applied to breast cancer cells (MCF7 and MDA-MB-231), lung cancer cells (A549), colorectal cancer cells (LoVo), and liver cancer cells (HA22T). DATS treatment induced a dose-dependent inhibition of cell viability in the cancer cells, with IC50 values of 94 µM ± 4.0 for MCF7, 84 µM ± 3.1 for MDA-MB-231, 78 µM ± 1.5 for A549, 72 µM ± 1.5 for LoVo, and 103 µM ± 1.5 for HA22T **(Figure 2B, Table 1)**.

These findings indicate that DATS is well-tolerated by non-cancerous cells, suggesting its potential as a complementary treatment to improve the chemosensitivity of cancer cells.

### 3.2 Diallyl trisulfide synergistically enhances doxorubicin cytotoxicity in breast cancer cells

Next, we investigated the potential of combining DATS with low doses of DOX to enhance chemosensitivity in breast cancer cells. Firstly, various concentrations of DOX (0.5, 1.0, 1.5, 2.0, and 2.5 μM) were treated to breast cancer cell lines MCF7 and MDA-MB-231 for 24 and 48 hours followed by MTT cell viability assay. Our results revealed that DOX treatment significantly induced a time and dose dependent inhibition of cell viability in breast cancer cells with IC_50_ values of 1.63 ± 0.15 μM in MCF7 cells and 2.1 ± 0.2 μM in MDA-MB-231 cells at 24 hours. The IC_50_ values at 48 hours were 0.9 ± 0.07 μM for MCF7 cells and 1.3 ± 0.25 μM for MDA-MB-231 cells, respectively **(Figure 2 A and 2B, Table 1)**.

Subsequently, the breast cancer cells were treated with increasing concentrations of DATS (25, 50, 75, 100, and 125 μM) for 24 and 48 hours. Similarly, DATS treatment significantly induced a time and dose dependent inhibition of cell viability. In MCF7 cells, DATS IC_50_ values were 94 ± 4 μM for 24 hours and 58 ± 2.5 μM for 48 hours. MDA-MB-231 cells were more sensitive to DATS, with IC_50_ values of 84 ± 3.1 μM for 24 hours and 49 ± 1.5 μM for 48 hours **(Figure 2C and 2D, Table 1)**.

To evaluate the synergistic potential of combining DATS with low doses of DOX, breast cancer cells were first treated with DOX (0.5 and 1 μM) for 24 hours, followed by the addition of DATS (25, 50, and 100 μM) for a combined treatment period of 48 hours. Our results revealed that DATS treatment significantly enhanced DOX chemosensitivity, effectively reducing the viability of both MCF7 and MDA-MB-231 cells **(Figure 2E and 2F, Supplementary Figure 1)**. Most combination pairs exhibited synergistic effects, as indicated by the combination index (CI < 1), except for the 0.5 and 1 μM DOX + 25 μM DATS pairs, which showed an additive effect **(Figure 2G and 2H)**. For subsequent experiments, we selected low doses of DOX (0.5 and 1 μM) and DATS (50 and 100 μM), as these combinations demonstrated strong synergy (CI < 0.7) and DATS at 50 and 100 μM had minimal toxicity to normal cells **(Figure 1)**. Taken together, these data demonstrate that Diallyl Trisulfide synergistically enhances doxorubicin cytotoxicity. Diallyl Trisulfide supplements could potentially improve chemotherapeutic responses in patients undergoing doxorubicin chemotherapy for breast cancer treatment.

### 3.3 Diallyl trisulfide and doxorubicin combination inhibits glucose metabolism in breast cancer cells

**Figure 3:**
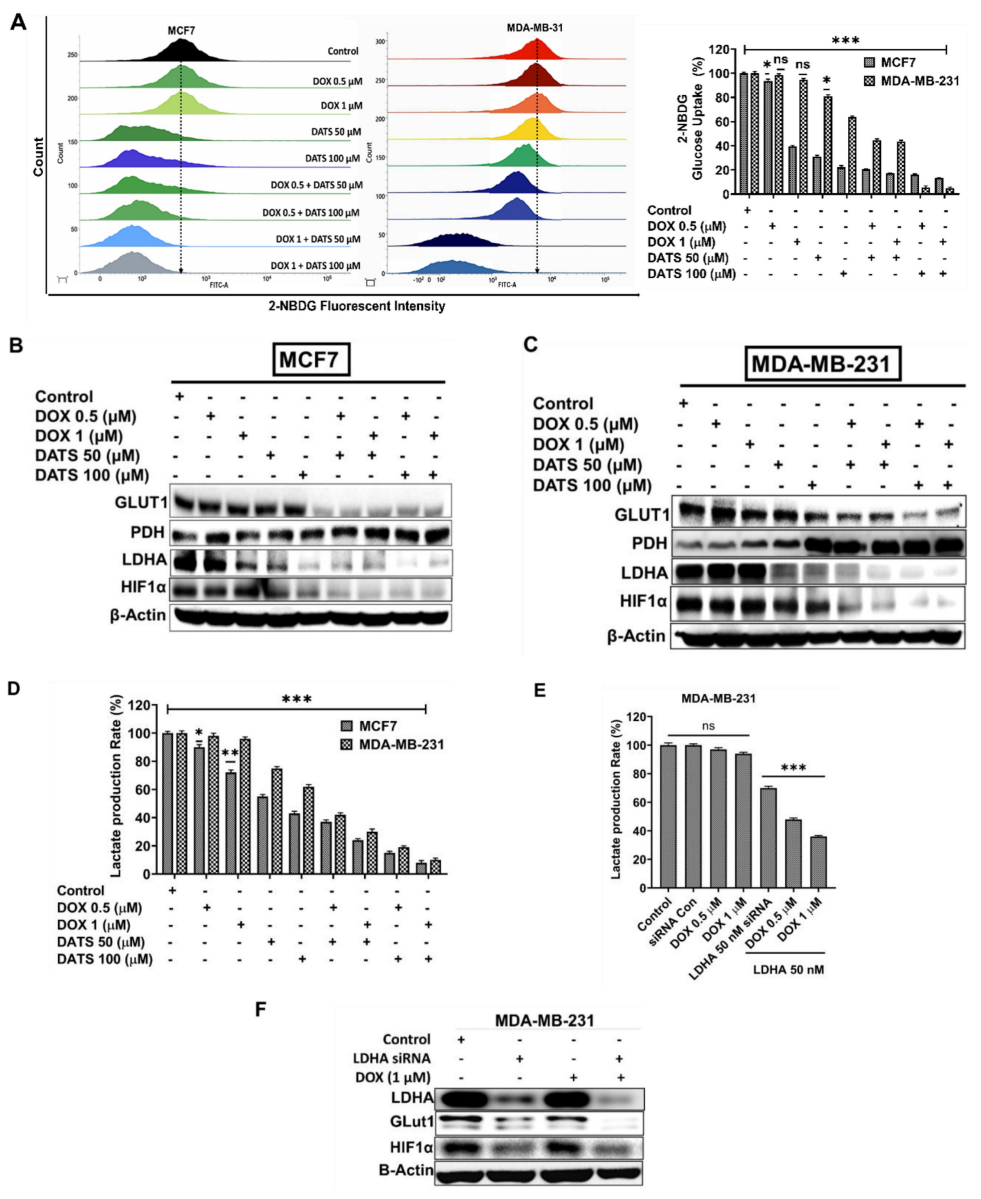
** Diallyl trisulfide and doxorubicin combination inhibits glucose metabolism in breast cancer cells. (A**) MCF7 and MDA-MB-231 breast cancer cells were treated with DOX (0.5 μM, 1 μM), DATS (50 μM, 100 μM), and their combination, followed by 2-NBDG glucose uptake flow cytometry analysis.** (B)** The same treatments as in **(A)** followed by western blot analysis of key glycolytic regulators: GLUT1, PDH, LDHA, and HIF-1α in MCF7 cancer cells. **(C)** Similar treatment as in **A** and immunoblotting as in **B** for MDA-MB-231 cells **(D)** Similar treatments as in **(A)** followed by a lactate production colorimetric assay. **(E)** Lactate production colorimetric assay in MDA-MB-231 cells exposed to LDHA-specific siRNA (50 nM) with or without Doxorubicin (0.5 and 1 µM). **(F)** Western blot analysis of LDHA, GLUT1 and HIF1α in MDA-MB-231 cells treated with LDHA-siRNA (50 nM) with or without DOX (1 µM). B-Actin is loading control. Data in panels (A), (D), and (E) are expressed as the mean ± SD of treated groups compared to the control, ns=not significant, *P < 0.05, **P < 0.01, ***P < 0.001.

We next examined the impact of DATS and DOX combination treatment on glucose metabolism in breast cancer cells. MCF7 and MDA-MB-231 cells were treated with DOX (0.5 and 1 μM) and DATS (50 and 100 μM), both individually and in combination, for 48 hours, followed by a glucose uptake assay. Flow cytometry analysis using the fluorescent glucose analog 2-NBDG revealed a significant reduction in glucose uptake in both cell lines following combination treatment compared to single-drug treatments **(Figure 3A)**.

To further explore the effects of DOX on glycolysis, MCF7 and MDA-MB-231 cells were treated with increasing concentrations of DOX (0.5, 1, 1.5, 2, and 2.5 μM), followed by Western blot analysis. The data demonstrated that DOX treatment had minimal impact on LDHA expression in MDA-MB-231 cells, in contrast to MCF7 cells, indicating that DOX alone was insufficient to inhibit lactate production in MDA-MB-231 cells at the tested concentrations **(Supplementary Figure 2)**.

Importantly, combination treatment remarkably suppressed key glycolytic regulators, including GLUT1, LDHA, and HIF-1α, while simultaneously enhancing the expression of pyruvate dehydrogenase (PDH) in both MCF7 and MDA-MB-231 cells **(Figures 3B and 3C)**. Consistent with these findings, lactate production was significantly reduced in response to the combination treatment in both cell lines **(Figure 3D)**.

To directly assess the role of LDHA in mediating these metabolic effects, LDHA-specific siRNA (50 nM) was used to knock down LDHA expression in MDA-MB-231 cells. Co-treatment with LDHA siRNA and DOX resulted in a marked decrease in lactate production **(Figure 3E)**. Furthermore, this combination also led to reduced expression of HIF-1α and GLUT1, further supporting the role of LDHA in regulating glycolytic activity in these cells **(Figure 3F)**.

Taken together, these data suggest that the anti-proliferative effects of DATS and DOX combination treatment may be mediated, at least in part, by suppression of glucose metabolism, highlighting a potential metabolic vulnerability in breast cancer cells.

### 3.4 Diallyl trisulfide and doxorubicin combination induces apoptosis in breast cancer cells

**Figure 4:**
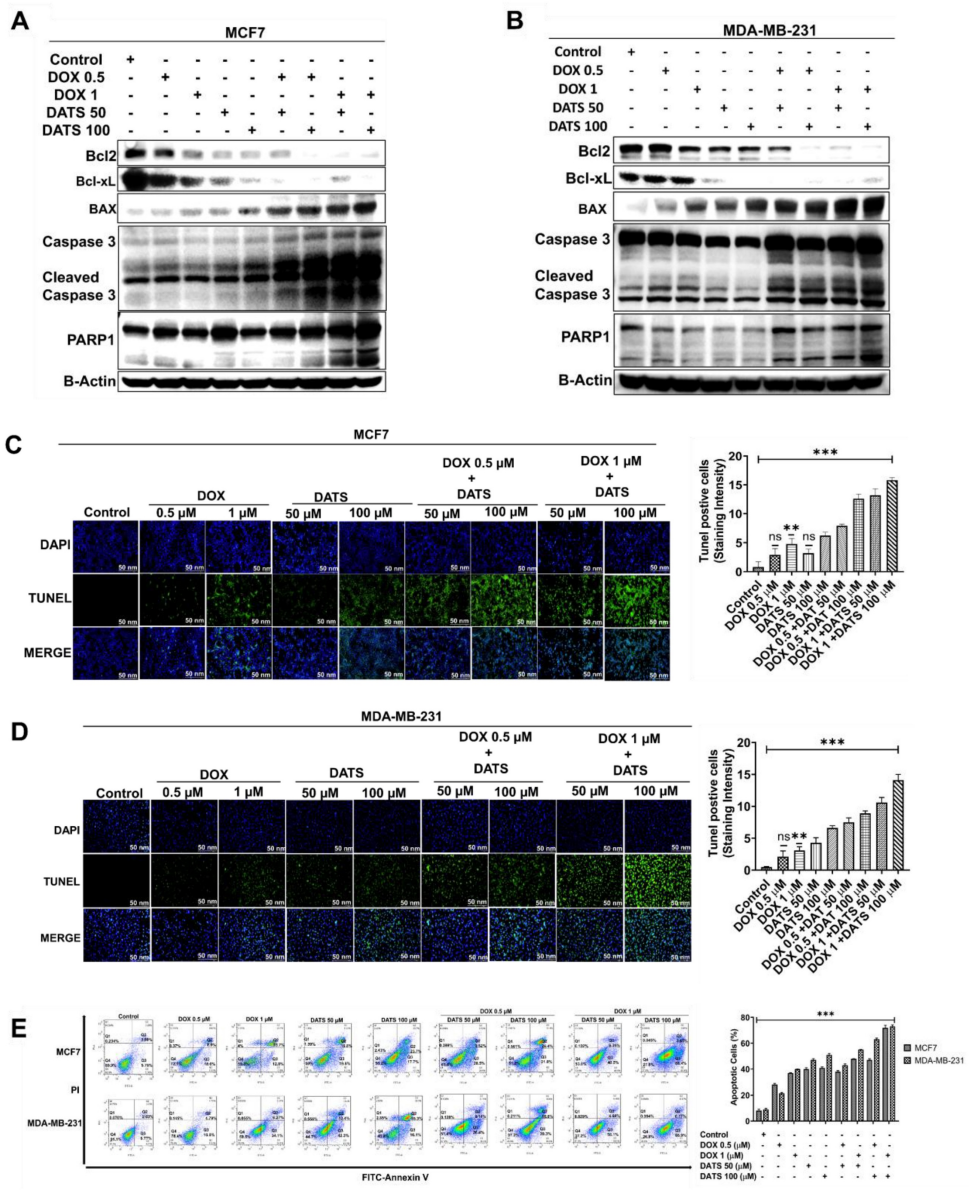
** Diallyl trisulfide and doxorubicin combination induces apoptosis in breast cancer cells. (A)** MCF7 cancer cells were treated with a combination of DOX (0.5 and 1 μM) and DATS (50 and 100 μM), followed by western blot analysis of the indicated apoptosis markers. **(B)** MDA-MB-231 cancer cells were treated similarly to (A), followed by western blot analysis. β-Actin was used as the loading control. **(C)** MCF7 cancer cells were treated similarly to (A), followed by TUNEL staining. **(D)** MDA-MB-231 cancer cells were treated similarly to (A), followed by TUNEL staining. **(E)** Annexin V-FITC/PI staining and flow cytometry analysis in MCF7 and MDA-MB-231 cells treated similarly to (A). Data in panels (C), (D), and (E) are expressed as the mean ± SD of treated groups compared to the control, ns=not significant, *P < 0.05, **P < 0.01, ***P < 0.001.

To investigate the pro-apoptotic effects of the DATS and DOX combination treatment, we assessed apoptotic marker expression in MCF7 and MDA-MB-231 breast cancer cells using western blot analysis. Cells treated with a combination of DOX (0.5 μM, 1 μM) and DATS (50 μM, 100 μM) exhibited a marked downregulation of the anti-apoptotic proteins Bcl-2 and Bcl-xL, accompanied by upregulation of the pro-apoptotic protein BAX. Furthermore, we observed enhanced cleavage of caspase-3 and PARP1, indicating activation of the intrinsic apoptotic pathway **(Figure 4A and 4B)**.

To further confirm apoptosis induction, we performed TUNEL assays, which demonstrated a significant increase in TUNEL-positive cells following DATS-DOX combination treatment in both cell lines **(Figure 4C and 4D)**. Additionally, Annexin V-FITC/PI double staining followed by flow cytometry analysis further validated apoptotic activation, as evidenced by an increased proportion of Annexin V-positive cells in the combination treatment group **(Figure 4E)**.

Together, these findings demonstrate that the combination of DATS and DOX synergistically induces apoptosis in breast cancer cells.

### 3.5 Diallyl trisulfide and doxorubicin combination inhibits mda-mb-231 breast cancer cell growth *in vivo*

**Figure 5:**
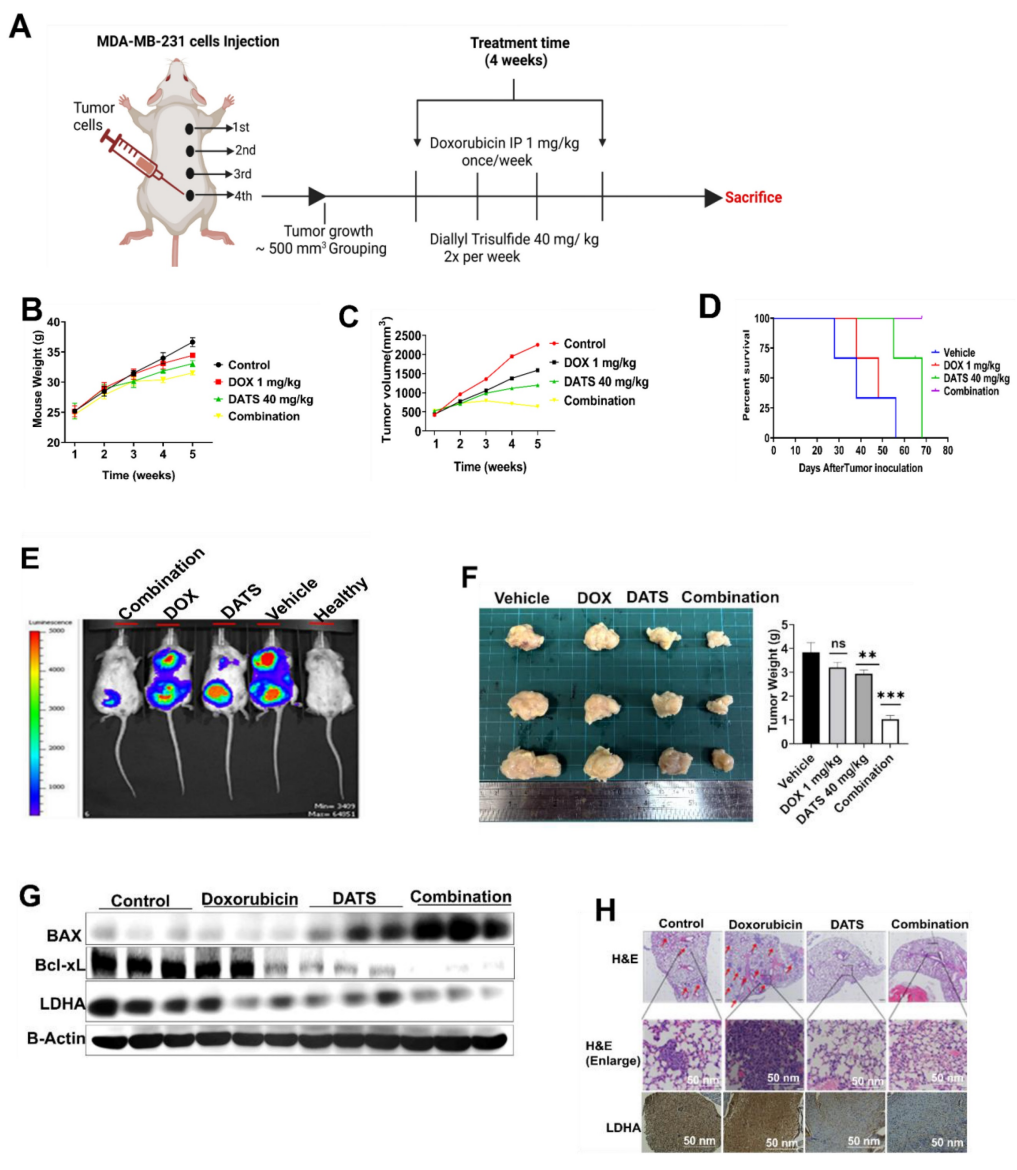
** Diallyl trisulfide and doxorubicin combination inhibits MDA-MB-231 breast cancer cell growth *in vivo*. (A)** Combination treatment *in vivo* experimental design. **(B)** Monitoring of mouse body weight during the treatment period. **(C)** Tumor volume monitoring during treatment period. (D) Kaplan-Meier survival analysis from tumor initiation to experiment end point. (E) Bioluminescence imaging of tumor-bearing mice at the end of the experiment. **(F)** Gross tumor size and weight from each group at the end of the experiment. **(G)** Western blot analysis of apoptotic markers (BAX, Bcl-xL) and metabolic markers (LDHA) in tumor tissue lysates, B-Actin is loading control. **(H)** H&E staining (upper panels) and LDHA immunohistochemistry (lower panels) in tumor sections. Representative images are shown, scale bar = 50 nm. Data are expressed as the mean ± SD of treated groups compared to the control, ns=not significant, **P < 0.01, ***P < 0.001.

To evaluate the therapeutic potential of the DATS and DOX combination treatment *in vivo*, luciferase-expressing MDA-MB-231 breast cancer cells were injected into the fourth mammary fat pad of NOD/SCID mice. The mice were randomly divided into four treatment groups when the tumor volume reached approximately 500 mm³: Vehicle, DOX (1 mg/kg, intraperitoneally once a week), DATS (40 mg/kg orally, twice a week), and the combination **(Figure 5A)**.

Monitoring of mouse body weight during the treatment period revealed no significant differences among the groups, indicating that the combination treatment did not induce overt toxicity in the mice **(Figure 5B)**. Importantly, the combination treatment significantly reduced tumor volume during the treatment period compared to single drug treatments and the control group **(Figure 5C)**. Kaplan-Meier survival analysis revealed that mice receiving the combination treatment showed significantly prolonged survival compared to all other groups **(Figure 5D)**.

At the experimental endpoint, bioluminescence imaging demonstrated reduced tumor luminescence signals in the combination treatment group compared to single drug treatments and the control **(Figure 5E)**. Gross tumor size and weight further confirmed the enhanced anti-tumor effects of the combination treatment **(Figure 5F)**.

Immunoblotting analysis confirmed the activation of apoptosis in the combination treatment group through enhanced expression of BAX and downregulation of Bcl-xL, as well as inhibition of glucose metabolism through suppression of LDHA **(Figure 5G)**. Hematoxylin and eosin (H&E) staining and LDHA immunohistochemistry confirmed reduced tumor burden and inhibition of glucose metabolism by the combination treatment **(Figure 5H)**.

Overall, these findings underscore the potential of DATS as a chemosensitizing agent that enhances the therapeutic efficacy of DOX in breast cancer cells *in vivo*.

## 4. Discussion

Breast cancer remains one of the most prevalent malignancies worldwide and a leading cause of cancer-related mortality in women 1. While doxorubicin remains a cornerstone of chemotherapy due to its potent anti-tumor activity, its clinical efficacy is frequently compromised by the emergence of chemoresistance and dose-limiting toxicity 6,7. Chemoresistance is often associated with metabolic reprogramming and evasion of apoptosis—two key mechanisms that drive tumor progression and poor treatment outcomes 10. Thus, identifying adjuvant compounds that enhance DOX efficacy while mitigating its adverse effects is of critical importance. Our study provides compelling evidence that diallyl trisulfide, a bioactive organosulfur compound derived from garlic, enhances DOX chemosensitivity by inhibiting the Warburg effect and promoting apoptosis in breast cancer cells.

A major challenge in cancer therapy is achieving selective cytotoxicity against malignant cells while sparing normal tissues. Our findings indicate that DATS exhibits preferential cytotoxicity toward breast cancer cells, with minimal toxicity in normal cells. Furthermore, our results demonstrate that the combination of DATS and DOX exerts a synergistic anti-cancer effect, as evidenced by a significant reduction in cell viability compared to monotherapy. These observations are consistent with previous studies showing that DATS selectively induces oxidative stress and mitochondrial dysfunction in cancer cells without significantly affecting normal cells 24. Additionally, organosulfur compounds have been reported to enhance the cytotoxic effects of conventional chemotherapeutic agents by modulating multiple oncogenic signaling pathways, reinforcing the therapeutic potential of DATS as a chemosensitizing agent 25,26.

Metabolic reprogramming is a hallmark of cancer, with the Warburg effect—a preference for aerobic glycolysis over oxidative phosphorylation—playing a crucial role in sustaining rapid tumor growth and mediating therapy resistance 9,10. Our study demonstrates that DATS disrupts glycolytic metabolism in breast cancer cells, as evidenced by a significant reduction in glucose uptake, downregulation of key glycolytic regulators (GLUT1, LDHA, and HIF1α), and diminished lactate production following combination treatment with DOX. These findings are in agreement with previous studies showing that organosulfur compounds suppress glycolysis by targeting critical metabolic enzymes, such as hexokinase-2 (HK2) and LDHA, thereby reducing ATP production and increasing oxidative stress 9,24,27-31. Given that metabolic plasticity is a major driver of doxorubicin resistance 32,33, the ability of DATS to inhibit glycolysis represents a promising strategy for overcoming chemoresistance. Notably, siRNA-mediated knockdown of LDHA further sensitized breast cancer cells to DOX, highlighting the pivotal role of glycolytic suppression in enhancing chemosensitivity. To further support this mechanism, molecular docking analysis revealed that DATS could occupy the substrate-binding pocket of LDHA, suggesting potential direct inhibition 34,35 (Supplementary Figure S3). Future studies involving comprehensive computational and experimental validation, including binding affinity assays, will be necessary to confirm this interaction and fully elucidate the molecular mechanisms involved.

In addition to metabolic inhibition, our study highlights the role of DATS in enhancing apoptosis in breast cancer cells. The combination of DATS and DOX led to a marked increase in pro-apoptotic markers (BAX, cleaved caspase-3, and PARP1) and a concurrent decrease in anti-apoptotic proteins (Bcl-2, Bcl-xL), as confirmed by TUNEL and Annexin V-FITC/PI assays. These findings suggest activation of the intrinsic apoptotic pathway and are in line with previous studies demonstrating that DATS induces apoptosis through multiple mechanisms, including mitochondrial dysfunction, caspase activation, and reactive oxygen species (ROS) generation 28,36-39. Furthermore, DATS has been reported to modulate key survival pathways, such as PI3K/Akt and NF-κB signaling, leading to downregulation of anti-apoptotic proteins such as Bcl2 and upregulation of pro-apoptotic factors like Bax 25,26,36,39-41. The disruption of these oncogenic pathways likely contributes to the enhanced chemosensitivity observed in our study.

The *in vivo* efficacy of DATS and DOX combination therapy was validated using an orthotopic MDA-MB-231 xenograft model. Tumor growth was significantly suppressed in the combination treatment group compared to monotherapies, with prolonged survival rates. Notably, these effects were associated with a marked reduction in LDHA expression, supporting the notion that metabolic inhibition contributes to tumor regression. Additionally, no significant changes in body weight were observed, suggesting that DATS does not exacerbate DOX systemic toxicity. These findings align with previous reports indicating that dietary organosulfur compounds can enhance the anti-tumor activity of chemotherapy while reducing its adverse effects 39,42.

In conclusion, our study provides compelling evidence that DATS enhances Dox chemosensitivity by inhibiting glycolysis and inducing apoptosis in breast cancer cells. The combination therapy demonstrated significant efficacy both *in vitro* and *in vivo*, supporting the potential of DATS as an adjuvant in breast cancer treatment. Future studies should explore the molecular mechanisms underlying this synergy and evaluate the clinical translation of DATS-based combination therapies.

## Supplementary Material

Supplementary figures.

## Figures and Tables

**Table 1:** IC_50_ values for DATS and DOX treated against MCF7, MDA-MB-231 and A549, LoVo, and HA22T cancer cells for the indicated times

	IC_50_ (µM)	SDTV	95% CI	
Cell/Treatment	Mean	(±)	Lower	Upper
MCF7 DOX 24 h	1.63	0.15	1.3	2.0
MCF7 DOX 48 h	0.9	0.07	0.7	1.0
MCF7 DATS 24h	94	4.0	84	104
MCF7 DATS 48 h	58	2.5	52	65
MDA-MB-231 DOX 24 h	2.1	0.20	1.6	2.6
MDA-MB-231 DOX 48 h	1.3	0.25	0.71	1.96
MDA-MB-231 DATS 24 h	84	3.1	76	91
MDA-MB-231 DATS 48 h	49	1.5	46	53
A549 DATS 24h	78	1.5	74	81
LoVo DATS 24h	72	1.5	69	76
HA22T DATS 24h	103	2	98	108
