# Supplementary material for: Diallyl Trisulfide Enhances Doxorubicin Chemosensitivity by Inhibiting the Warburg Effect and Inducing Apoptosis in Breast Cancer Cells
Source: J Cancer. 2025 Jul 11;16(11):3283–95. doi: 10.7150/jca.113578 (PMC12374837; doi:10.7150/jca.113578)
Supplement: Supplementary file 1 — Supplementary figures. [file jcav16p3283s1.pdf]

A
B
C

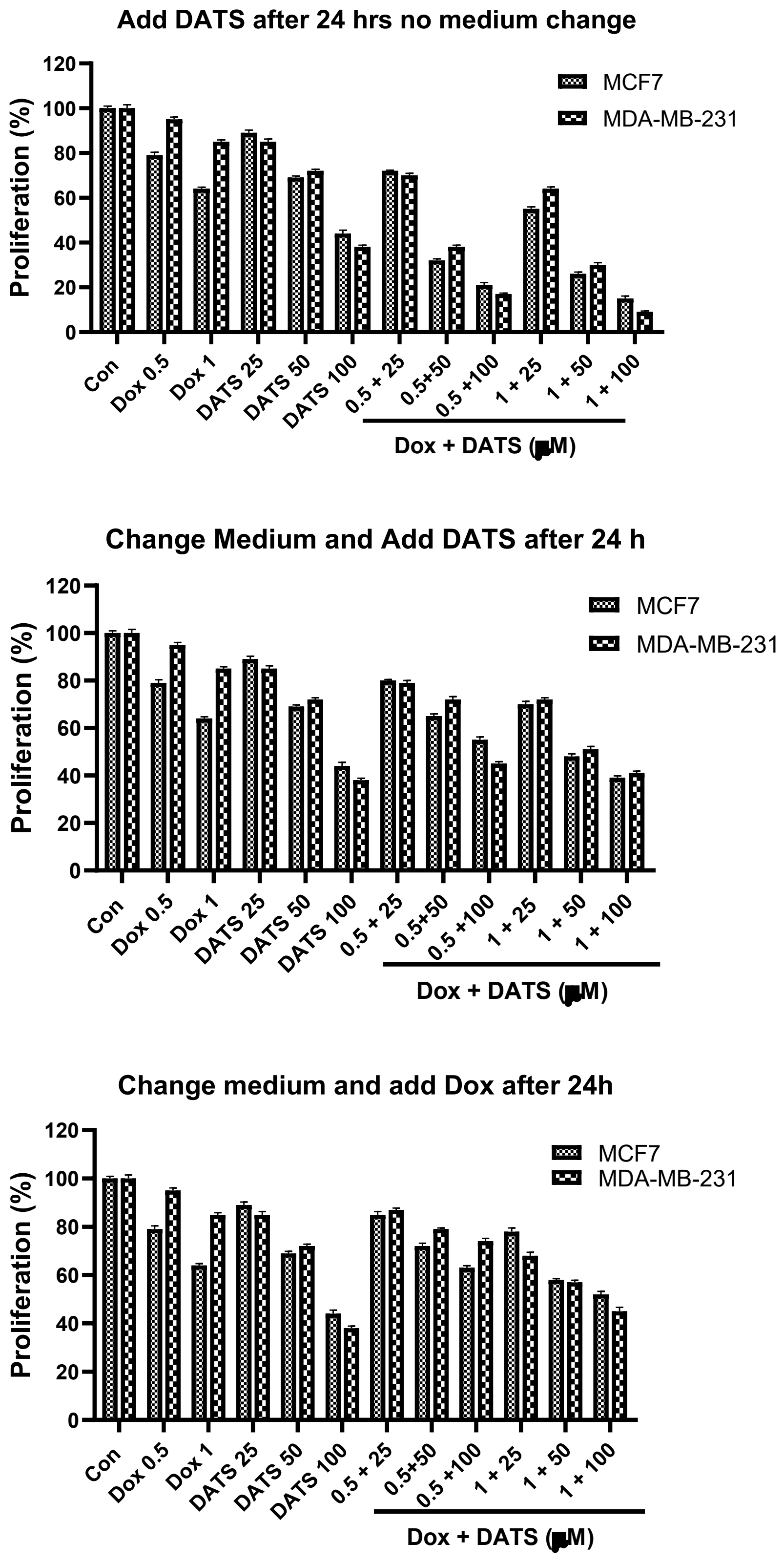

Add DATS after 24 hrs no medium change
Proliferation (%)
0
20
40
60
80
100
120
MCF7
MDA-MB-231
Con
Dox 0.5
Dox 1
DATS 25
DATS 50
DATS 100
0.5 + 25
0.5+50
0.5 +100
1 + 25
1 + 50
1 + 100
Dox + DATS (μM)
Change Medium and Add DATS after 24 h
Proliferation (%)
0
20
40
60
80
100
120
MCF7
MDA-MB-231
Con
Dox 0.5
Dox 1
DATS 25
DATS 50
DATS 100
0.5 + 25
0.5+50
0.5 +100
1 + 25
1 + 50
1 + 100
Dox + DATS (μM)
Change medium and add Dox after 24h
Proliferation (%)
0
20
40
60
80
100
120
MCF7
MDA-MB-231
Con
Dox 0.5
Dox 1
DATS 25
DATS 50
DATS 100
0.5 + 25
0.5+50
0.5 +100
1 + 25
1 + 50
1 + 100
Dox + DATS (μM)

Supplementary Figure 1: Evaluation of the Synergistic Potential of Diallyl Trisulfide and Doxorubicin Combination Treatment

(A) MCF7 and MDA-MB-231 breast cancer cells were co-treated with 0.5 μM or 1 μM Doxorubicin (Dox). After 24 hours, the indicated concentrations of Diallyl Trisulfide (DATS) were added without changing the culture medium. Cell viability was assessed using the MTT assay. **(B)** MCF7 and MDA-MB-231 cells were treated as in (A), but the culture medium was replaced before adding the indicated concentrations of DATS. Cell viability was subsequently evaluated using the MTT assay. **(C)** MCF7 and MDA-MB-231 cells were pre-treated with DATS at the indicated concentrations. After replacing the culture medium, cells were treated with the indicated concentrations of Dox, followed by MTT assay to determine cell viability. The results are presented as the percentage of viable cells in treated groups relative to control cells treated with DMSO. These experiments provide insights into the optimal sequence and conditions for combining DATS and Dox to achieve synergistic cytotoxicity against breast cancer cells.

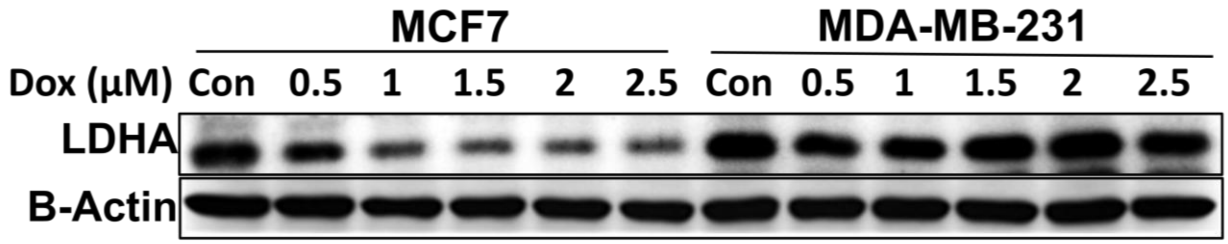

Supplimentaty Figure 2. Doxorubicin Treatment did not affect LDHA expression in MDA-MB-231 breast cancer cells. LDHA Immunoblotting in DOX treated MCF7 and MDA-MB-231 breast cancer cells. DOX treatment alone did not significantly alter LDHA protein expression when compared to MCF-7 cancer cells. (β-actin is loading control)

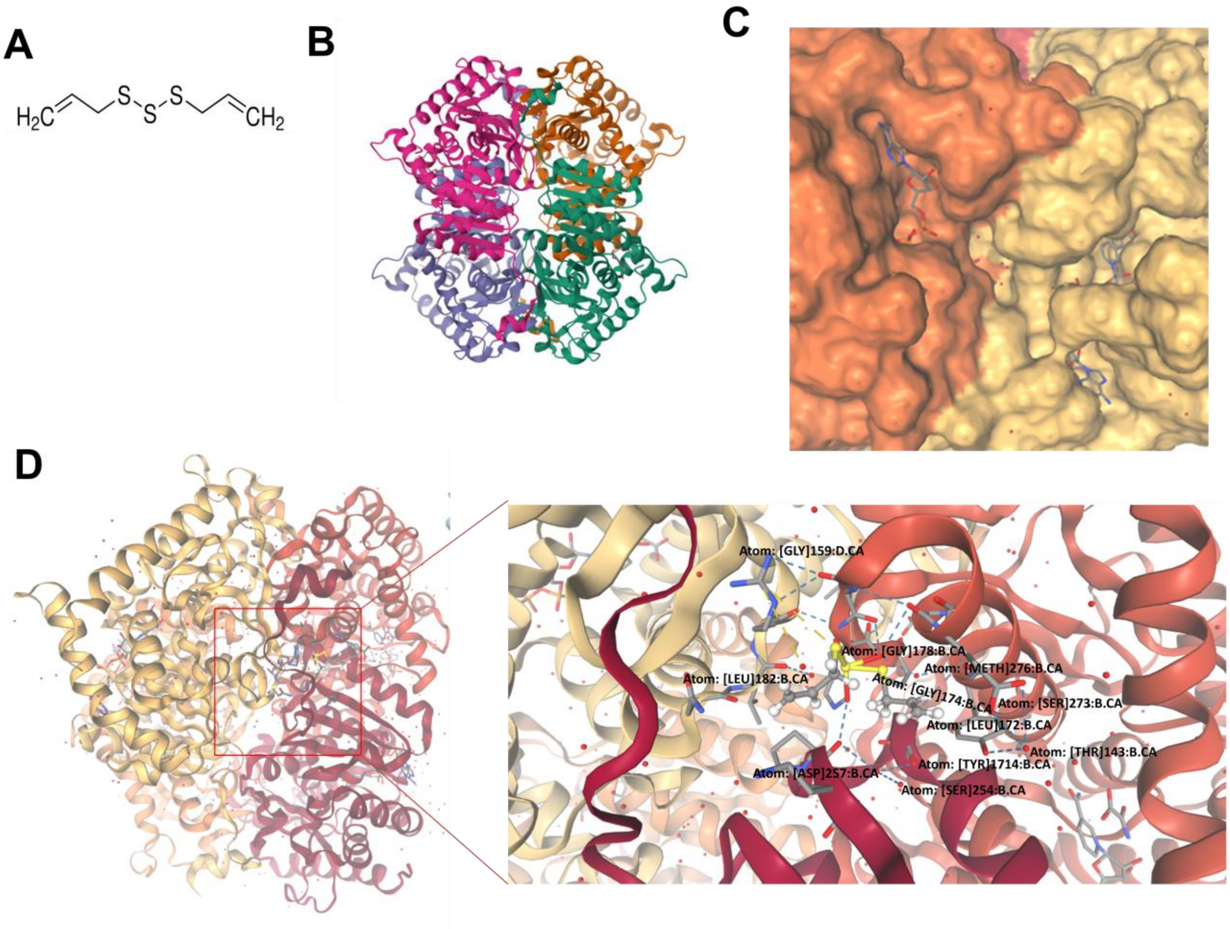

Supplementary Figure 3. Pose analysis visualization of the top-scoring Diallyl trisulfide docking pose within the active site of LDHA.

(A) Chemical structure of Diallyl trisulfide. **(B)** Ribbon representation of the LDHA tetramer highlighting its quaternary structure, with each monomer colored distinctly. **(C)** Surface representation of the LDHA active site showing the binding pocket, with DATS docked within the interface of two subunits (orange and yellow surfaces). **(D)** Detailed view of the binding interactions between DATS and LDHA. The zoomed-in panel highlights critical amino acid residues in proximity to DATS, including GLY159, GLY178, LEU182, MET176, SER273, LEU272, THR143, TYR174, ASP257, and SER254. Hydrogen bonds, ionic interactions, hydrophobic interactions are depicted, suggesting potential stabilization of DATS within the active site. The visualization illustrates that DATS effectively occupies the substrate-binding pocket of LDHA, suggesting a possible mechanism of inhibition through direct binding.
